# History of Coleoptera collecting in New Brunswick, Canada: advancing our knowledge of the Coleoptera fauna in the early 21^st^ century

**DOI:** 10.3897/zookeys.573.8123

**Published:** 2016-03-24

**Authors:** Reginald P. Webster, Patrice Bouchard, Jan Klimaszewski, Jon D. Sweeney

**Affiliations:** 1 24 Mill Stream Drive, Charters Settlement, NB, Canada E3C 1X1; 2 Agriculture and Agri-Food Canada, Canadian National Collection of Insects, Arachnids and Nematodes, Ottawa, Ontario, Canada K1A 0C6; 3 Natural Resources Canada, Canadian Forest Service - Laurentian Forestry Centre, 1055 du P.E.P.S., P.O. Box 10380, Stn. Sainte-Foy, Québec, Quebec, Canada G1V 4C7; 4 Natural Resources Canada, Canadian Forest Service - Atlantic Forestry Centre, 1350 Regent St., P.O. Box 4000, Fredericton, NB, Canada E3B 5P7

The Coleoptera of New Brunswick have generated interest among entomologists for over a century. The first records of Coleoptera from New Brunswick were the adventive *Carabus granulatus* Linnaeus and *Carabus nemoralis* Müller collected by W.H. Harrington in Saint John during 1891 (Harrington 1892). The first significant sampling of Coleoptera, and insects in general in New Brunswick, was carried out by members of the Natural History Society of New Brunswick (now the New Brunswick Museum): William McIntosh, Phillip R. McIntosh, A. Gordon Leavitt, and George Morrisey, mostly between 1898 and 1909 (Fairweather and McAlpine 2011). Most of the material was obtained by William McIntosh and A. Gordon Leavitt, who made extensive collections around the Saint John area (Fairweather and McAlpine 2011). By 1914, there were over 24,000 specimens in the Natural History Society of New Brunswick insect holdings, most being Lepidoptera, with about 4,187 specimens of Coleoptera (McIntosh [undated A]). However, only 1,095 of these Coleoptera specimens were still present in the New Brunswick Museum (NBM) holdings in 2010, many apparently were either sent to other people or were lost to insect pests (Fairweather and McAlpine 2011). Among these specimens are the first occurrences of a number of adventive species to the Maritime provinces: *Quedius mesomelinus* (Marsham) (Staphylinidae) (Majka and Smetana 2007), *Attagenus unicolor japonicas* Reitter (Dermestidae) (Majka 2007a), *Ernobius mollis* (Linnaeus) (Ptiliidae) (Majka 2007a), *Brachypera zoilus* (Scopoli) (Curculionidae) (Majka et al. 2007b), and others, including many that were the first records for New Brunswick and the region.

In subsequent years, J.N. Knull from Ohio State University visited New Brunswick during 1927 and collected beetles, C.A. Frost of the Cambridge Entomological Club of Massachusetts collected insects, including beetles, between 1926 and 1930 in Penobsquis, and W.J. Brown collected beetles in northern and eastern New Brunswick from 1926 to 1943 (Majka and Johnson 2008). Many Coleoptera were collected from the 1920s through 1940s by R.P. Gorham of the Dominion Entomological Laboratories. Another significant source of Coleoptera specimens was the Forest Insect and Disease Survey (FIDS) of the Canadian Forest Service (CFS) in Fredericton. This survey began in 1936 and was discontinued in 1996. Most of these specimens are in the collection at the Atlantic Forestry Centre (AFC) in Fredericton and the Canadian National Collection of Insects, Arachnids and Nematodes (CNC) in Ottawa. During the summer months of 1977 and 1978, scientists from the Biosystematics Research Center (currently the Ottawa Research and Development Centre, Agriculture and Agri-Food Canada) collected Coleoptera during a survey of the invertebrate fauna at Kouchibouguac National Park. A report of this major undertaking summarized the most significant findings in the families Carabidae, Dytiscidae, Staphylinidae, Silphidae, Scarabaeidae, Lampyridae, Coccinellidae, and Chrysomelidae (Miller and Lyons 1979). Many of the beetle specimens from Kouchibouguac National Park were subsequently included in revisions of Canadian Coleoptera. During 1987 and 1988, A. Larochelle and M.C. Larivière surveyed the Carabidae of Maine and the Maritime Provinces and reported 64 species new to New Brunswick (Larochelle and Larivière 1990). Many of the records from the above collections and surveys were included in the first *Checklist of the beetles of Canada and Alaska*, where 1,365 species were reported as occurring in New Brunswick (Bousquet 1991).

Since the 1970s, students and staff at the Université de Moncton have been collecting beetles, mostly in the eastern areas of the province; these specimens are currently housed in that collection (UMC). In 2003, Anne-Sophie Bertrand, then a graduate student at the Université de Moncton, collected Carabidae and other Coleoptera, including three species of Carabidae new to the province, as part of a study focused on biological indicators of old-growth forests in northwestern New Brunswick (Bertrand 2005). Gaétan Moreau and Martin Turgeon also collected beetles in northwestern New Brunswick and provided a number of new records in recent years. Donald F. McAlpine at the NBM, began collecting insects and beetles in the early 1990s in the Grand Bay area. This material is housed in the NBM. Later, starting in 2009, McAlpine, in an effort to address deficiencies in knowledge of the biodiversity of New Brunswick’s Protected Natural Areas, organized a series of broad-based, volunteer-supported biological inventories (Bioblitzes) at the Jacquet River Gorge Protected Natural Area (PNA), the Caledonia Gorge PNA, and the Grand Lake Lowlands PNA (McAlpine 2011). A significant number of Coleoptera specimens were collected during these surveys, many of which were new provincial records and species new to science. A significant number of beetles were collected between 1992 and 1995 in Fredericton during a study examining vertical and temporal distribution of Carabidae and Elateridae above potato fields, including several species of Elateridae and Carabidae, new to New Brunswick (Boiteau et al. 2000). In another study organized by Jon Sweeney (Natural Resources Canada (NRCan) CFS - AFC) that used pitfall traps to investigate the effects of silvicultural practices on diversity and abundance of ground beetles in red spruce stands, 58 new provincial records and seven species of Staphylinidae new to science were discovered (Klimaszewski et al. 2005). Many Coleoptera from other families, particularly Carabidae, that were collected during this study are in the AFC collection. Reginald Webster conducted additional surveys at these same sites during 2007 as part of a follow-up study examining changes in diversity influenced by succession. Christopher G. Majka began collecting Coleoptera in Albert Co. in 1965 and continues to sample this area (Majka and Johnson 2008). Majka examined various regional collections, including the NBM, UMC, CNC, and many other collections and published a series of papers between 2005 and 2011, reviewing the Coleoptera fauna of the Maritime Provinces, which included numerous new records from New Brunswick. Majka and various coauthors treated 61 families (listed in taxonomic order) in the following publications, adding 259 new records for New Brunswick, including eight new Canadian records, and one new North American record [number of new provincial records (NPR) or new Canadian records (NCR) in brackets]: Gyrinidae [4 NPR] (Majka and Kenner 2009), Carabidae [6 NPR] (Majka et al. 2007c), Haliplidae [6 NPR] (Majka et al. 2009d), Histeridae [2 NPR] (Majka 2008a), Ptiliidae [5 NPR including 1 NCR] (Majka and Sörensson 2007), Leiodidae [8 NPR] (Majka and Langor 2008), Lucanidae [3 NPR] (Majka 2008c), Eucinetidae [1 NPR] (Majka 2010a), Clambidae [2 NPR] Majka and Langor 2009), Byrrhidae [4 NPR] (Majka et al. 2007d, Majka and Langor 2011b), Eucnemidae [1 NPR] (Majka 2007c), Throscidae (Majka 2011b), Elateridae [13 NPR] (Majka and Johnson 2008), Derodontidae, Dermestidae [3 NPR], Bostrichidae [1 NPR], Ptinidae [3 NPR] (Majka 2007b), Trogossitidae [3 NPR] (Majka 2011c), Cleridae [3 NPR] (Majka 2006b), Melyridae [3 NPR] (Majka 2005), Sphindidae [2 NPR] (Majka 2010b), Erotylidae [2 NPR] (Majka 2007a, Majka et al. 2010c), Monotomidae [1 NPR] (Majka and Bousquet 2010), Cryptophagidae [9 NPR including 1 NCR] (Majka et al. 2010a, Majka and Langor 2010), Silvanidae [2 NPR], Cucujidae [1 NPR], Laemophloeidae [2 NPR] (Majka 2008b), Phalacridae [2 NPR] (Majka et al. 2008b), Kateretidae [3 NPR], Nitidulidae [28 NPR] (Majka et al. 2008d), Cerylonidae (Majka and Langor 2011c), Endomychidae [2 NPR] (Majka 2007a, 2009), Coccinellidae (Majka and McCorquodale 2006), Corylophidae [2 NPR] (Majka and Cline 2006), Latridiidae [11 NPR including 4 NCR and 1 new North American record] (Majka et al. 2009a), Mycetophagidae [3 NPR] (Majka 2010c), Tetratomidae, Melandryidae [3 NPR] (Majka and Pollock 2006), Mordellidae [6 NPR], Ripiphoridae [1 NPR] (Majka and Jackman 2006), Zopheridae (Majka et al. 2006), Tenebrionidae [13 NPR] (Majka et al. 2008a), Stenotrachelidae [1 NPR] (Majka 2011a), Oedemeridae [2 NPR] (Majka and Langor 2011a), Boridae [1 NPR], Pythidae [1 NPR], Pyrochroidae, Salpingidae [1 NPR] (Majka 2006a), Anthicidae [3 NPR] (Majka and Ogden 2006, Majka 2011d), Aderidae (Majka 2011e), Scraptiidae (Majka and Pollock 2006), Ischaliidae [1 NPR] (Majka and Ogden 2006), Cerambycidae [2 NPR] (Majka et al. 2010b), Chrysomelidae [2 NPR] (Majka and LeSage 2007, 2008a, 2008b, 2010, Majka and Kirby 2011), Nemonychidae [1 NPR], Anthribidae [3 NPR], Attelabidae, Brentidae [6 NPR including 1 NCR], Dryophthoridae [2 NPR], Brachyceridae [2 NPR], Curculionidae [67 NPR including 2 NCR] (Majka et al. 2007a, 2007b, 2008c).

Reginald Webster began intensively sampling beetles in New Brunswick in the early 1990s, initially focusing on the Carabidae, but later broadening his efforts to the Dytiscidae and other families in the early 2000s. Sampling, using a variety of methods such as sifting litter, hand sampling, sweeping, and light trapping, was done throughout the province, but was concentrated in the Fredericton and Charters Settlement area in York Co. and the Grand Lake area in Queens Co. Between 2006 and 2008, Webster in partnership with Stephen Clayden, NBM Curator of Botany, examined the beetle and lichen communities of old-growth New Brunswick cedar stands. This work revealed numerous species among both groups that were new to the region or that were new to science and led directly to the protection of several sites under the provincial Protected Natural Areas Act (McAlpine 2011). Webster also conducted surveys at the Daly Point Reserve in Bathurst, the Stillwater watershed area near Kedgwick in Restigouche Co., the Portobello Creek (Sunbury and Queens Co.) and Shepody National Wildlife (Albert Co.) areas, and the Meduxneakeag Valley Nature Preserve and the Bell Forest in Carleton Co. In a study led by Jon Sweeney (NRCan, AFC) to develop improved methods for survey and detection of exotic and potentially invasive bark and wood-boring beetles (Cerambycidae, Buprestidae, Curculionidae), many Coleoptera specimens were collected in Lindgren funnel traps. Vincent Webster, Chantelle Alderson, Colin MacKay, Marie-Andrée Giguère, Cory Hughes, Michelle Roy, and others collected and processed many of those samples. Experiments were conducted between 2009 and 2015 at sites throughout the province in most forest types, often in old or old-growth stands in Protected Natural Areas.

Webster and various coauthors, published a series of papers between 2008 and 2012 on new records from the province, based on the above sampling efforts. Fifty-nine families were treated (listed in taxonomic order) in the following publications, adding 448 new records for New Brunswick, including nine new Canadian records: Gyrinidae [2 NPR] (Webster and DeMerchant 2012a), Carabidae [54 NPR] (Webster and Bousquet 2008, Webster and DeMerchant 2012a) Dytiscidae [19 NPR including 1 NCR] (Webster 2008, Webster and DeMerchant 2012a); Histeridae [18 NPR] (Webster et al. 2012e); Geotrupidae [2 NPR], Scarabaeidae [12 NPR] (Webster et al. 2012g); Eucinetidae [2 NPR], Scirtidae [5 NPR including 1 NCR] (Webster et al. 2012h); Buprestidae [9 NPR] (Webster and DeMerchant 2012b); Dryopidae [1 NPR], Elmidae [1 NPR], Psephenidae [2 NPR], Ptilodactylidae [1 NPR] (Webster and DeMerchant 2012c); Eucnemidae [9 NPR] (Webster et al. 2012i); Elateridae [22 NPR] (Webster et al. 2012j); Lycidae [8 NPR] (Webster et al. 2012k); Dermestidae [2 NPR], Endecatomidae [1 NPR], Bostrichidae [2 NPR], Ptinidae [5 NPR] (Webster et al. 2012x); Trogossitidae [2 NPR], Cleridae [1 NPR], Melyridae [2 NPR] (Webster et al. 2012l); Silvanidae [2 NPR], Laemophloeidae [3 NPR] (Webster et al. 2012m); Sphindidae [2 NPR], Erotylidae [5 NPR], Monotomidae [3 NPR], Cryptophagidae [6 NPR] (Webster et al. 2012n); Kateretidae [1 NPR], Nitidulidae [3 NPR], Cerylonidae [2 NPR], Endomychidae [2 NPR], Coccinellidae [3 NPR], Latridiidae [8 NPR] (Webster et al. 2012o); Mycetophagidae [4 NPR], Tetratomidae [7 NPR], Melandryidae [10 NPR] (Webster et al. 2012p); Mordellidae [11 NPR including 1 NCR], Ripiphoridae [1 NPR] (Webster et al. 2012q); Tenebrionidae [13 NPR], Zopheridae [2 NPR] (Webster et al. 2012v); Stenotrachelidae [1 NPR], Oedemeridae [2 NPR], Meloidae [3 NPR including 1 NCR], Mycteridae [1 NPR], Boridae, Pythidae [1 NPR], Pyrochroidae [3 NPR], Anthicidae [5 NPR], Aderidae [3 NPR] (Webster et al. 2012r); Cerambycidae [52 NPR including 4 NCR] (Webster et al. 2009b, Webster et al. 2012w); Megalopodidae [1 NPR], Chrysomelidae [28 NPR] (Webster et al. 2012d); Anthribidae [3 NPR], Brentidae [4 NPR], Dryophthoridae [3 NPR], Brachyceridae [3 NPR], Curculionidae [50 NPR including 1 NCR] (Webster et al. 2012a). In these papers, new habitat and biological data were presented for many of the species. Smetana and Webster (2011) described *Quedius bicoloris* Smetana and Webster, based in part on specimens from New Brunswick. Douglas et al. (2013) reported another four species of Anthribidae, one new Brentidae, and 11 new Curculionidae from the province. Revisions by Hieke (2000, 2003) added three species of *Amara* (Carabidae) to the provincial list. Dwayne Sabine reported the rare *Cicindela marginipennis* Dejean for the first time from Canada from New Brunswick (Sabine 2004) and Bousquet and Webster (2006) described *Bembidion iridipenne* Bousquet and Webster and *Bembidion nigrivestris* Bousquet, in part from specimens collected in New Brunswick.

The Staphylinidae of New Brunswick received relatively little attention prior to the publication of the first edition of the “Checklist of the beetles of Canada and Alaska”. Only 166 species of Staphylinidae, including 19 species in the subfamily Aleocharinae, were recorded from the province by Campbell and Davies (1991). In the Aleocharinae alone, Reginald Webster and coauthors Jan Klimaszewski, Christopher Majka, and others published a series of papers between 2001 and 2012 that included 183 new records from New Brunswick. Among these were 29 new Canadian records and 15 species new to science (NS), described, in many cases, from material from New Brunswick. These generic treatments are as follows: *Placusa* [1 NPR] Klimaszewski et al. 2001), *Tinotus* (1 NCR) (Klimaszewski et al. 2002), *Silusa* [2 NPR including 1 NCR)](Klimaszewski et al. 2003), *Leptusa* [1 NS] (Klimaszewski et al. 2004), *Oxypoda* [4 NPR] (Klimaszewski et al. 2006), *Atheta acadiensis* Klimaszewski and Majka [1 NS] (Klimaszewski and Majka 2007), *Amarochara* [1 NPR (NCR), 1 NS] (Assing 2007), *Calodera* [1 NPR (NCR)] (Assing (2008), *Gnypeta* [3 NPR including 2 NCR, 2 NS] (Klimaszewski et al. 2008a), *Diglotta* and *Halobrecta* [2 NPR (NCR)] Klimaszewski et al. 2008b), *Schistoglossa* [1 NPR, 2 NS] (Klimaszewki et al. 2009a), *Gyrophaena* [19 NPR including 8 NCR, 2 NS], *Eumicrota* [2 NPR] (Klimaszewski et al. 2009b), *Alisalia* [1 NPR (NCR), 2 NS] (Klimaszewski et al. 2009c), *Aleochara sekanai* Klimaszewski [1 NPR] (Majka and Klimaszewski 2009), Aleocharinae [28 NCR including 5 NCR, 4 NS] (Klimaszewski et al. 2005), Aleocharinae [3 NPR including 1 NCR which was also new to North America] (Klimaszewski et al. 2007), Aleocharinae [12 NPR] (Majka and Klimaszewski 2008a, b, c, 2010), Aleocharinae [86 NPR including 6 NCR] (Webster et al. 2009a, 2012c).

In other subfamilies of Staphylinidae, Chris Majka contributed 19 new records for New Brunswick in the following papers: introduced species [10 NPR] (Majka and Klimaszewski 2008a), adventive species [4 NPR] (Majka and Klimaszewski 2008d), adventive *Quedius* [2 NPR], Majka and Smetana 2007, *Quedius cinctus* (Paykull) (NCR) (Majka et al. 2009b), *Quedius spelaeus* Horn [NPR] (Majka and Brown 2010), *Philonthus hepaticus* Erichson (NPR) (Majka et al. 2009c). Webster and coauthors newly recorded an additional 156 staphylinid species from New Brunswick, including one new Canadian record, from the following subfamilies: Omaliinae [11 NPR], Micropeplinae [2 NPR], Phloeocharinae [1 NPR], Olisthaerinae [1 NPR], Habrocerinae [3 NPR] (Webster et al. 2012s), Pselaphinae [20 NPR including 2 NCR] (Webster et al. 2012b), Tachyporinae [33 NPR including 1 NCR] (Webster et al. 2012t), Scaphidiinae [9 NPR], Piestinae [2 NPR], Osoriinae [2 NPR], Oxytelinae [6 NPR] (Webster et al. 2012u), Oxyporinae [5 NPR] (Webster and DeMerchant 2012d), Paederinae [17 NPR] (Webster and DeMerchant 2012e), and Staphylininae [44 NPR] (Webster et al. 2012f).

**Figure 1.**
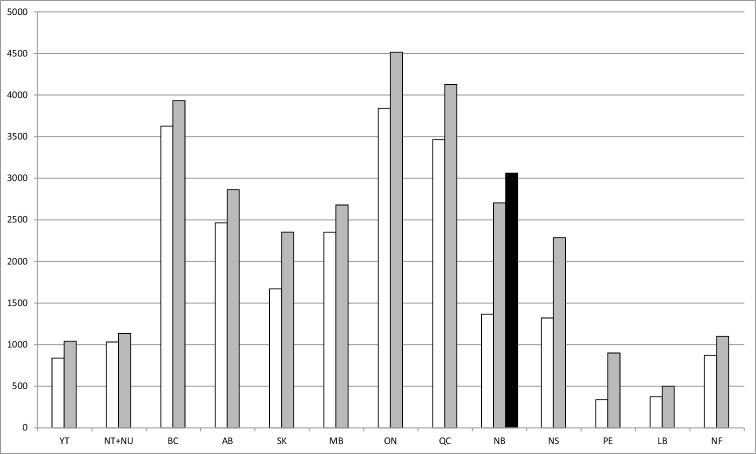
Number of species recorded for Canadian provinces and territories over time. Data extracted from: Bousquet (1991), white bars; Bousquet et al. (2013), gray bars; this article, black bar. Acronyms: AB: Alberta, BC: British Columbia, LB: Labrador, MB: Manitoba, NB: New Brunswick, NF: Newfoundland, NS: Nova Scotia, NT: Northern Territory, NU: Nunavut, ON: Ontario, PE: Prince Edward Island, QC: Quebec, SK: Saskatchewan, YT: Yukon Territory.

As a result of the above publications and additional data from material in the CNC, the number of species reported from New Brunswick nearly doubled from the 1,365 species reported in the first *Checklist of the beetles of Canada and Alaska* by Bousquet (1991) to 2,703 species in the latest checklist by Bousquet et al. (2013) (Fig. 1). This is a significant change in our knowledge of the Coleoptera fauna of New Brunswick.

Since the publication of Bousquet et al. (2013) and prior to this current special issue of Zookeys, an additional 53 species have been added to the faunal list of New Brunswick as a result of new species descriptions and new records in recent publications. Klimaszewski et al. (2013, 2014, 2015b, c) added 19 species of Aleocharinae in the genera *Atheta*, *Clusiota*, *Dinaraea*, *Gnathusa*, *Mniusa*, *Ocyusa*, and *Mocyta* to the faunal list of New Brunswick, based on new species descriptions and new records. Puthz (2014), in a review of North American species of *Euaesthetus* (Staphylinidae, Euaesthetinae) added nine species to the provincial list, including three that were new to science, based in part on material collected in New Brunswick. Makranczy (2014) in his revision of the *Ochthephilus* (Oxytelinae), described *Ochthephilus ashei* Makanczy, based in part on a specimen from NB, and reported *Ochthephilus forticornis* (Hochhuth) and *Ochthephilus planus* (LeConte) from the province, both of which were new provincial records. Bousquet and Bouchard (2014), in a review of the *Paratenetus* of North America, described *Paratenetus exutus* Bousquet and Bouchard (Tenebrionidae) from New Brunswick and included many localities from the province. *Carabus auratus auratus* Linnaeus (Carabidae) was newly recorded for Canada from New Brunswick by Lewis et al. (2015), and *Buprestis consularis* Gory (Buprestidae) was added by Lewis (2015). *Dryocoetes krivolutzkajae* Mandelshtam (Curculionidae) was reported for the first time for North America by Cognato et al. (2015), in part, from specimens from New Brunswick. Klimaszewski et al. (2015a) newly recorded the adventive *Cryptophagus saginatus* Sturm and *Cryptophagus subfumatus* Kraatz (Cryptophagidae) in a review of the adventive Cucujoidea of Canada. Most recently, Webster et al. (2016) newly reported 16 species of Cerambycidae.

In this special issue, 303 species and one new subspecies are newly recorded for New Brunswick. Among the new records are 32 species new to science, four new North American records, 21 new Canadian records, 270 new provincial records, and 45 adventive species. Three species were removed from the provincial list and one species was re-instated that was erroneously not included for New Brunswick by Bousquet et al. (2013). This brings the total number of species known from New Brunswick to 3,062. This is a 13% increase in the number of species listed for New Brunswick since Bousquet et al. (2013) and a 124% increase since the publication of Bousquet (1991) (Fig. 1).

It is important to remind ourselves that the understanding of biological diversity is not possible without taxonomic research, which is thought by many to be the foundation of biological science. Data on the mega-diversity of life and knowledge on species identity and distribution require discovery, description, cataloguing, and organization in order to be made accessible to a wide audience. This information constitutes a baseline of biological knowledge that is critical to support other branches of science. The present work provides these baseline data for the Coleoptera occurring in New Brunswick. This work would not have been possible to complete without the enthusiasm, determination, and professionalism of a small number of dedicated individuals who are acknowledged in the papers in this special issue. We hope that this special issue will generate a positive response and further interest in the Coleoptera fauna of New Brunswick and Canada, as many new discoveries await.
